# Is Pelvic Plexus Block Superior to Periprostatic Nerve Block for Pain Control during Transrectal Ultrasonography-Guided Prostate Biopsy? A Double-Blind, Randomized Controlled Trial

**DOI:** 10.3390/jcm8040557

**Published:** 2019-04-24

**Authors:** Do Kyung Kim, Yoon Soo Hah, Jong Won Kim, Kyo Chul Koo, Kwang Suk Lee, Chang Hee Hong, Byung Ha Chung, Kang Su Cho

**Affiliations:** 1 Department of Urology, Soonchunhyang University Seoul Hospital, Soonchunhyang University Medical College, Seoul 04401, Korea; dokyung80@hotmail.com; 2 Department of Urology, Daegu Catholic University Medical Center, Daegu 42472, Korea; uro.drhah@gmail.com; 3 Department of Urology, Gangnam Severance Hospital, Urological Science Institute, Yonsei University College of Medicine, Seoul 06273, Korea; doctor2play@yuhs.ac (J.W.K.); gckoo@yuhs.ac (K.C.K.); calmenow@yuhs.ac (K.S.L.); chhong52@yuhs.ac (C.H.H.); chung646@yuhs.ac (B.H.C.)

**Keywords:** prostate, biopsy, transrectal, ultrasound, local anesthesia

## Abstract

We evaluated whether pelvic plexus block (PPB) is superior to periprostatic nerve block (PNB) for pain control during transrectal ultrasonography (TRUS)-guided prostate biopsy (PBx). A prospective, double-blind, randomized, controlled study was performed at a single center; 46 patients were enrolled and randomly allocated into two groups: PPB (*n* = 23) and PNB (*n* = 23). The visual analogue scale (VAS) was used; pain scores were measured four times: during local anesthesia, probe insertion, sampling procedures, and at 15 min post procedures. No significant differences were observed in VAS scores during local anesthesia (2.30 for PPB vs. 2.65 for PNB, *p* = 0.537) or during probe insertion (2.83 for PPB vs. 2.39 for PNB, *p* = 0.569). Similarly, no differences in VAS scores were detected during the sampling procedures (2.83 for PPB vs. 2.87 for PNB, *p* = 0.867) and at 15 min post procedures (1.39 for PPB vs. 1.26 for PNB, *p* = 0.631). No major complications were noted in either group. Both PPB and PNB are comparably effective and safe methods for PBx related pain relief, and PPB is not superior to PNB. Local anesthetic method could be selected based on the preference and skill of the operator.

## 1. Introduction

Prostate cancer (PCa) is the most frequently occurring malignancy in men worldwide, with approximately 1.1 million new cases diagnosed each year [1]. Transrectal ultrasonography (TRUS)-guided prostate biopsy (PBx) is the gold standard for detection of PCa. The current standard PBx, using 10–14 biopsy cores, detects PCa in about 44% of patients, and it is estimated that 2.5 million procedures are performed each year worldwide [2]. Notably, patients undergoing PBx are under considerable psychological stress due to the potential for a cancer diagnosis, fear of anal penetration, and the pain induced by both probe insertion and core biopsy [3].

Recently, various local anesthetic methods have been developed to reduce PBx-related pain. These involve multiple types of anesthetic agents and sites of injection, and when used either alone or in combination, most were found to be effective for control of PBx-related pain [4]. During periprostatic nerve block (PNB), the most commonly used method, lidocaine is injected into the bilateral junctions between the bladder, prostate, and seminal vesicle, with the intent to block the posterolateral neurovascular bundle that is responsible for supplying the main nerve to the prostate [5,6,7,8,9,10].

Pelvic plexus block (PPB), which involves injecting lidocaine directly lateral to the tip of the seminal vesicles, was first described by Wu et al. [11]. Because the pelvic plexus is located more proximally, it has been hypothesized that PPB may result in a wider block area than a localized block in the periprostatic area. Thus, PPB may be more effective for alleviating PBx-related pain, as compared to PNB [12]. Accordingly, several investigators have concluded that PPB is more effective than PNB through prospective randomized controlled trials (RCTs) [12,13,14]. However, the number of such studies is relatively small, making it difficult to draw solid conclusions in terms of reproducibility. In addition, PPB is not widely used in common clinical practices. For these reasons, we aimed to determine whether PPB is superior to PNB for pain control during TRUS-guided PBx in this study.

## 2. Materials and Methods

### 2.1. Study Design

This prospective, double-blind, randomized, controlled study was performed at one institution (Gangnam Severance Hospital) from September 2018 to February 2019. Ethical approval was obtained from the Institutional Ethical Committee (Approval No.: 3-2018-0211), and the study is registered at http://clinicaltrials.gov (clinicaltrials.gov ID: NCT03681522). 

### 2.2. Eligibility Criteria for Participants

Patients with an abnormal prostate finding during digital rectal examination (DRE), a serum prostate-specific antigen (PSA) level >2.5 ng/mL, and/or an abnormal lesion detected with TRUS were included in this study. Those under 50 years of age or with a history of previous TRUS-guided biopsy, chronic prostatitis/pelvic pain, neurological conditions (dementia, Parkinson’s disease, or cerebral infarction, etc.), bleeding diathesis, anticoagulation/antiplatelet therapy, active urinary tract infection, hemorrhoids/anal fissure/anal fistula, or a known allergy to lidocaine were excluded from the study. Before the biopsy procedure, patients with active urinary tract infection and prostatitis were excluded by investigation of current symptoms and past history and urine analysis. Written informed consent was obtained from all patients prior to study enrollment.

### 2.3. Sample Size Estimation and Randomization

Using pain scores reported in the literature [12], we estimated the mean and standard deviation for pain scores from PPB to be 4.97 and 2.16, respectively, and the mean and standard deviation for the pain scores from PNB to be 2.7 and 1.95, respectively. Based on this, the number of samples required to achieve 95% power and 5% probability of type-1 error was 23 subjects per group. We estimated the dropout rate to be 20% and recruited 29 people for each group. Sample size calculation was performed using G*Power software [15].

Enrolled patients were randomly allocated by computer randomization into the PPB and PNB groups. The former received PPB and the latter received PNB. 

### 2.4. Technique for Biopsy Procedures

All patients took levofloxacin (500 mg, once daily) prior to the procedure, for a total of five days, and received intravenous third-generation cephalosporine on the day of the procedure, according to the institutional protocol. A cleansing enema was also performed on the morning of biopsy. All block procedures were performed by a single well-experienced urologist who was accustomed with five years of experience with performing the block procedure. Another single urologist, with more than seven years of prostate biopsy experience, conducted all the biopsy procedures using a BK 3000 scanner (BK medical, Herlev, Denmark), with a 4- to 14-MHz endorectal triplane transducer. During the procedure, patients were placed in a right lateral decubitus position. EMLA cream (lidocaine 2.5% and prilocaine 2.5%; Astrazeneca, UK) was applied first around the anal ring and 10 mL 2% lignocaine jelly was instilled into rectum 10 min before the introduction of the TRUS probe. Nerve block (either PPB or PNB), with 2.5 mL 2% lidocaine, was injected into both sides of the prostate (5 mL total), using a 21 G, 15 cm Chiba needle (A & A M.D. Inc., Seongnam, Korea). 

In the PPB group, lidocaine injections were administered to the pelvic neurovascular plexus, located at the end of the seminal vesicle, under Doppler-guided ultrasound, on each side [11,12,13,14]. In the PNB group, lidocaine injection was administered into the neurovascular bundles at the junction of the prostate–bladder–seminal vesicle, on each side, as described in previous studies [5,7,8,9,10,14]. After 5 min, a 12-core biopsy, covering the base, mid-zone, and apex bilaterally, was taken in all patients using an 18 G, 20 cm Max-Core disposable core biopsy instrument (CR Bard Inc., Covington, GA, USA).

### 2.5. Blinding and Assessment

All study participants were blinded to their treatment group assignment, and the urologist who conducted the biopsy procedures was blinded as to the type of nerve block given. A physician’s assistant, who was also not aware of the type of nerve block administered, explained the visual analog scale (VAS) to the participants and requested they rate their degree of pain or discomfort from 0 to 10 at four time points: (1) VAS-1, during the local anesthesia procedure, (2) VAS-2, during ultrasound probe insertion, (3) VAS-3, during the sampling procedure, and (4) VAS-4, 15 min after finishing all procedures. In addition, the physician’s assistant recorded the time it took to complete all procedures. The patients recorded VAS scores themselves, without assistance. The patients were observed for 4 h after the procedures and were counseled about the possibilities of mild hematuria, self-limiting rectal bleeding, mild fever, and/or hematospermia. They were advised to go to the emergency room if they experienced a high fever, syncope, massive hematuria, or urinary retention. All patients were reviewed again after one week to monitor for any late complications.

### 2.6. Statistical Methodology

The primary outcome of the present study was VAS scores at the time of biopsy. Pain scores (VAS-1, -2, -3, and -4) and the time to complete all procedures were compared between the two groups using the Mann–Whitney test. *p*-values were calculated using SPSS version 23 (SPSS Inc., Chicago, IL, USA), and *p* < 0.05 was considered statistically significant. 

## 3. Results

### 3.1. Patient Flow, Recruitment, and Number Analyzed

**Figure 1:**
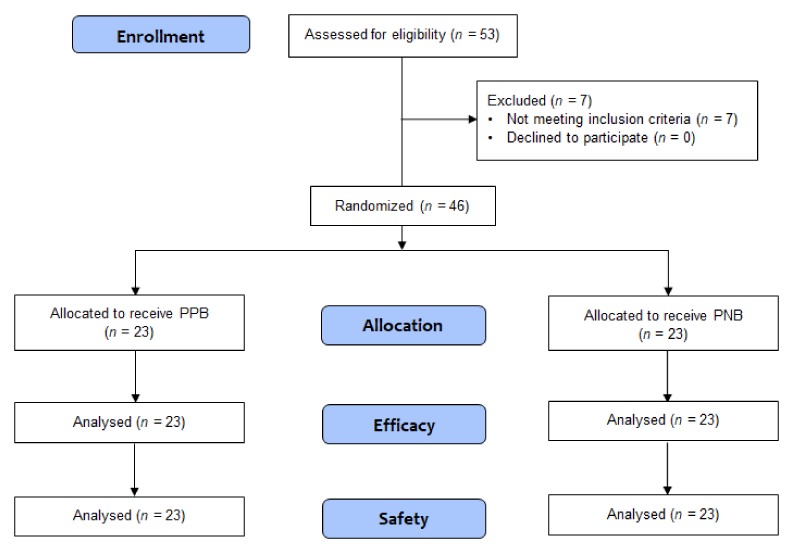
Consolidated standard of reporting trials flow diagram of study participants. PPB, pelvic plexus block; PNB, periprostatic nerve block.

Overall, 53 patients were assessed for eligibility and provided consent to be included in the study. Of these, seven patients did not meet the inclusion criteria (Figure 1): four had undergone a previous biopsy procedure, two had neurological conditions that made it difficult to understand and communicate for pain assessment (i.e., Parkinson’s disease and cerebral infarction), and one showed pyuria in urinalysis. The study was discontinued when the number of patients enrolled met the minimum requirement for statistical analysis (*n* = 46). These 46 patients were randomized into two groups, PPB (*n* = 23) and PNB (*n* = 23).

### 3.2. Outcomes

There were no significant differences in age, prostate volume, and serum PSA between the two treatment groups. The total time for all procedure was 12.52 ± 3.39 min for the PPB group and 13.78 ± 2.91 min for the PNB group (*p* = 0.154) (Table 1). Table 2 shows the mean VAS pain scores at four time points for each group, and we detected no significant difference in pain scores at any time point. Specifically, mean pain scores during the local anesthetic procedure (VAS-1) were 2.30 ± 1.52 for the PPB group and 2.65 ± 1.79 for the PNB group (*p* = 0.537). Mean pain scores at ultrasound probe insertion after anesthetic procedure (VAS-2) were 2.83 ± 2.21 for the PPB group and 2.39 ± 1.90 for the PNB group (*p* = 0.569). There were also no differences in mean pain scores during the sampling procedure (VAS-3; 2.83 ± 2.03 with PPB vs. 2.87 ± 1.93 with PNB, *p* = 0.867), and for those measured 15 min after finishing all procedures (1.39 ± 1.47 with PPB vs. 1.26 ± 1.48 with PNB, *p* = 0.631). 

### 3.3. Complications

There were no major complications, including urinary tract infection, sepsis, or significant rectal or urethral bleeding, reported by any of the patients. 

## 4. Discussion

Pain during PBx is caused by the introduction and manipulation of the ultrasound probe into the anal canal and rectum, as well as by penetration of the needle into the prostate capsule, which is rich in autonomic sympathetic and parasympathetic fibers [16,17]. In general, PBx is an ambulatory procedure, therefore a variety of local anesthetic methods have been attempted to reduce PBx-related pain. Although there is no perfect method for controlling pain during PBx, ongoing research has focused on determining the most effective method for alleviating PBx-related pain. Nash et al. were the first to describe the use of PNB for PBx pain relief in 1996 [16], and there is strong evidence that injection of local anesthetics into the periprostatic tissue decreases pain during this procedure. Consistent with this, meta-analyses of previous RCTs have reported that PNB is associated with significantly less pain than either placebo or no injection [3,4,18].

PPB was first described as an alternative to PNB by Wu et al. [11] in 2001 and involves injecting lidocaine directly lateral to the tip of the seminal vesicles under gray-scale ultrasonography guidance. The pelvic plexus is an autonomic plexus, composed of sympathetic and parasympathetic fibers, the midpoint of which lies lateral to the tip of the seminal vesicle [19]. It was hypothesized that the anatomy of the pelvic plexus may result in a wider pain block than a localized block in the periprostatic area [12]. Therefore, when lidocaine is injected bilaterally, directly into the pelvic plexus during PPB (thereby blocking all the nerve fibers proximally), this may have a better effect on pain control than PNB [14]. However, Wu et al. [11] failed to show a significant PPB-mediated reduction in PBx-related pain compared to the placebo arm. Subsequently, other researchers have conducted RCTs comparing the efficacy of PPB with that of PNB and have reported a significantly lower pain score in the PPB group during sampling of the prostate [12,13,14]. In these studies, unlike Wu et al. [11], Doppler ultrasound was used to improve the accuracy of injection into the pelvic plexus, and accordingly, Cantinello et al. [13] suggested that the more precise localization of the pelvic plexus by use of Doppler ultrasound might be the reason for the inconsistent findings. 

Recent meta-analysis by Li et al. [4] found that PPB has a better effect on pain control than PNB. However, the number of past studies addressing this question is relatively small, making it difficult to draw solid conclusions in terms of reproducibility, and the Grading of Recommendations, Assessments, Developments, and Evaluation (GRADE) assessment of the direct comparison between the two groups showed a low level of certainty of evidence [20]. This suggests that further research is highly likely to have a significant impact on their confidence in estimating the effects and changes in estimates. In addition, PPB is not widely used in common clinical practice. Thus, for all of these reasons, we performed the current study to confirm whether PPB is superior to PNB for pain control during TRUS-guided PBx. Here, unlike in former studies, we found that both PPB and PNB are comparably effective and safe methods for PBx-related pain relief, and that PPB is not superior to PNB. 

The characteristics and outcomes of this present study, as compared to previously published studies, are summarized in Table 3. In our study, mean VAS score during the sampling procedure in the group receiving PPB was 2.83, which is comparable to those reported in past studies (i.e., 2.7 in Akpinar et al. [12], 2.28 in Cantinello et al. [13], and 2.9 in Jindal et al. [14]). However, our mean VAS score during the sampling procedure in the PNB group was 2.87, which is considerably lower than scores from the aforementioned studies (i.e., 4.97 in Akpinar et al. [12], 3.37 in Cantinello et al. [13], and 4.0 in Jindal et al. [14]). This may be due to the difference in the proficiency of the performer or the same person in the nerve block and biopsy procedure. Based on these differences of result, we conclude that PPB is not superior to PNB, which is an important basis for further study.

We conducted a study of 46 patients; 23 across two groups. The total number of participants may be considered too small when compared to the previous studies [12,13,14]. However, estimation of the projected sample size of medical research is based on the hypothesis proposed by the researcher. If the investigator fails to determine the sample size before the start of the study for any reason, they should estimate the number of participants based on experience, logistics or something even more unscientific [21]. Moreover, research studies with too many participants potentially exposes the subjects to unnecessary risk and uses excess resources [22]. Therefore, studies should be conducted with a minimum number of subjects to prevent participants from being exposed to unnecessary risks. Thus, we calculated the number of samples based on the previous study that effect of PPB for controlling pain associated with PBx is superior than PNB [12]. Therefore, the calculated number of samples was appropriate to confirm the superiority of PPB compared with PNB.

Notably, all studies being compared were designed as double-blind investigations; however, we speculate that the current study may have less bias in the blinding step, due to the fact that clinicians performing the anesthesia, the biopsy procedure, and the access of the VAS scores were different and the physicians performing PBx and accessing the VAS score were blinded to the type of local anesthetic method. Other differences include the fact that Akpinar et al. [12] performed only PPB and PNB without intrarectal lidocaine gel instillation, whereas the others utilized PPB and PNB in combination with intrarectal lidocaine gel instillation. In addition, Cantinello et al. [13] conducted PPB and PNB with 1% lidocaine and 0.75% naropin, whereas the others performed the procedure with 2% lidocaine, and different amounts of anesthetic were used for the procedure in various studies (2 mL vs. 2.5 mL on each side). Further, the time intervals between anesthesia and sampling were either 5 min (in the present study and Cantinello et al. [13]) or 10 min (Jindal et al. [14]). In contrast, 12 cores were biopsied in all studies, and in each case, PNB was injected at the base. Jindal et al. [14] only reported one major complication (high-grade fever requiring hospitalization) in the PNB group; the others reported that there were no major complications. Both the present study and Cantinello et al. [13] found no significant differences in pain scale scores for PNB vs. PPB and detected no difference in the total time for PBx between the two methods.

We note, however, that the present study was not designed to evaluate the pain scores based on the site of biopsy, and therefore it remains unclear whether PPB may help in controlling pain associated with apex biopsies, due to the blocking of the anterior nerve fibers that might be missed by PNB. Further studies will thus be needed to clarify this issue. Lastly, VAS is widely used for epidemiological and clinical studies, and its strengths and weaknesses are well appreciated [23]. In particular, patients often have difficulty in determining the point that best describes the pain they feel [24,25]. Thus, the VAS scores reported can be higher or lower than results obtained from actually talking to patients and asking them to describe the pain they are experiencing. Moreover, the interpretation of a single VAS may be subjective [26]. Critically the VAS is not a precise scientific and reproducible tool, but there currently is not a better way to compare the anesthetic effect during PBx.

## 5. Conclusions

We found that despite the theoretical advantage of PPB, this technique did not show superiority for PBx-related pain control as compared to PNB. Thus, our data suggest that both PPB and PNB are comparably effective and safe methods for pain control in patients undergoing PBx, and as such, the local anesthetic method utilized in this procedure could be selected based on the preference and skill of the operator.

## Figures and Tables

**Table 1:** Patient characteristics in the PPB and PNB treatment groups.

Variable	All	PPB Group	PNB Group	*p*-Value
Number of patients (*n*)	46	23	23	
Age, years	68.5 ± 5.71	67.35 ± 1.28	69.65 ± 1.07	0.261
Prostate volume, mL	37.98 ± 15.76	37.8 ± 2.88	38.14 ± 3.71	0.692
PSA level, ng/mL	9.33 ± 10.14	8.47 ± 1.53	10.18 ± 2.59	0.886
Procedure time, min	13.15 ± 3.19	12.52 ± 3.39	13.78 ± 2.91	0.154

PNB, periprostatic nerve block; PPB, pelvic plexus block; PSA, prostate-specific antigen; data are presented as mean ± standard deviation.

**Table 2:** Mean VAS pain scores and procedure times in PPB and PNB treatment groups.

Pain Scores	All	PPB Group	PNB Group	*p*-Value
VAS-1: local anesthetic procedure	2.48 ± 1.66	2.30 ± 1.52	2.65 ± 1.79	0.537
VAS-2: probe insertion	2.61 ± 2.05	2.83 ± 2.21	2.39 ± 1.90	0.569
VAS-3: sampling procedures	2.85 ± 1.98	2.83 ± 2.03	2.87 ± 1.96	0.867
VAS-4: 15 min post procedures	1.33 ± 1.46	1.39 ± 1.47	1.26 ± 1.48	0.631

PNB, periprostatic nerve block; PPB, pelvic plexus block; VAS, visual analogue scale; data are presented as mean ± standard deviation.

**Table 3:** Characteristics of the present study and previously published studies comparing PPB and PNB.

Study	Study Design	Treatment Arms (Number of Patients)	Age (Mean ± SD)	Anesthetic Methods (PPB and PNB)	Intrarectal Lidocaine Gel Instillation	Number of Biopsy Cores	Injection Site of PNB	Time Interval between Anesthesia and Sampling	Sampling VAS (Mean ± SD)	Complications
Present study	RCT, double-blind ^†^	(1) PPB (23) (2) PNB (23)	(1) PPB: 67.35 ± 1.28 (2) PNB: 69.65 ± 1.07	2% lidocaine (2.5 mL on each side)	Yes	12	Base	5 min	(1) PPB: 2.85 ± 1.98 (2) PNB: 2.87 ± 1.96	No major complications
Akpinar et al. [12]	RCT, double-blind ^‡^	(1) PPB (40) (2) PNB (40)	(1) PPB: 64.9 ± 9.3 (2) PNB: 61.7 ± 9.5	2% lidocaine (2 mL on each side)	No	12	Base	NA	(1) PPB: 2.7 ± 1.95 (2) PNB: 4.97 ± 2.16	No major complications
Cantinello et al. [13]	RCT, double-blind ^‡^	(1) PPB (90) (2) PNB (90)	(1) PPB: 63.7 ± 5.4 (2) PNB: 63.2 ± 5.5	1% lidocaine + 0.75% naropin (2.5 mL on each side)	Yes	12	Base	5 min	(1) PPB: 2.28 ± 0.84 (2) PNB: 3.37 ± 0.78	No major complications
Jindal et al. [14]	RCT, double-blind ^‡^	(1) PPB (47) (2) PNB (46)	(1) PPB: 68.7 ± 9.1 (2) PNB: 67.3 ± 9.5	2% lidocaine (2.5 mL on each side)	Yes	12	Base	10 min	(1) PPB: 2.9 ± 0.84 (2) PNB: 4.0 ± 0.85	PNB group: high-grade fever (1)

PNB, periprostatic nerve block; PPB, pelvic plexus block; RCT, randomized controlled trial; SD, standard deviation; VAS, visual analogue scale. † Clinicians performing the anesthesia, the biopsy procedure, and the access of the VAS scores were different and the physicians performing PBx and accessing VAS score were blinded to the type of local anesthetic method; ‡ Clinicians performing the anesthesia and accessing the VAS scores were different and the physician accessing VAS score was blinded to the type of local anesthetic method.
